# The Effectiveness of Topical Fluorides in Preventing Dental Caries in Children and Adolescents: A Systematic Review and Meta-Analysis

**DOI:** 10.3290/j.ohpd.c_2826

**Published:** 2026-09-29

**Authors:** Meijia Shao, Qinfen Shen, Wenjun Zhao

**Affiliations:** a Meijia Shao Dental nurse, Department of Endodontics, Shaoxing Stomatological Hospital, Shaoxing, 312000, China. Study design, conducting the study, analysing the data, writing and reviewing the manuscript.; b Qinfen Shen Dentist, Department of Endodontics, Shaoxing Stomatological Hospital, Shaoxing, 312000, China. Study design, conducting the study, analysing the data, writing and reviewing the manuscript.; c Wenjun Zhao Dental nurse, Department of Endodontics, Shaoxing Stomatological Hospital, Shaoxing, 312000, China. Study design, conducting the study, analysing the data, writing and reviewing the manuscript.

**Keywords:** caries prevention, children and adolescents, meta-analysis, safety, topical fluoride

## Abstract

**Objective:**

This study aims to assess the effectiveness of topical fluoride treatments, including mouth rinses, toothpaste, gels, foams and varnish, in preventing dental caries in children and adolescents through a systematic review and meta-analysis. It primarily focuses on the impact of different intervention frequencies, durations, concentrations, and types of fluoride on the incidence of dental caries and on changes in decayed, missing and filled surfaces (DMFS) and the surface area of initial caries (SIC) indices.

**Methods and Materials:**

Clinical intervention studies that met the inclusion criteria were identified through searches of PubMed, Embase, Web of Science, Cochrane Library, ScienceDirect, SinoMed, Chinese National Knowledge Infrastructure and Wanfang databases. The literature search was conducted on 2 June 2025 and covered publications between 1 January 2000 and 31 May 2025. Two independent reviewers performed literature screening, data extraction and risk-of-bias assessment. A meta-analysis was performed using fixed-effects or random-effects models. Subgroup analyses and narrative heterogeneity exploration were conducted according to fluoride concentration, intervention frequency, duration, age group and control type. Safety outcomes were narratively summarised.

**Results:**

The meta-analysis of 10 included clinical studies showed that topical fluoride significantly reduced caries incidence when the event was defined as development of dental caries (odds ratio [OR] = 0.27, 95% CI: 0.21–0.36, *p* < 0.00001), prevented DMFS increment (weighted mean difference [WMD] = 0.35, 95% CI: 0.07–0.63, *p* = 0.01) and reduced SIC changes (WMD = −0.85, 95% CI: −1.66 to −0.05, *p* = 0.04). Subgroup analyses indicated superior outcomes with 0.2% fluoride, higher intervention frequency and longer duration. The 5% sodium fluoride varnish study was summarised separately because it used a professionally applied varnish and delayed-varnish control. Safety reporting was limited, and no serious fluoride-related adverse events were identified in the available reports. Formal meta-regression was not performed because the number of study-level estimates for DMFS and SIC was limited.

**Conclusion:**

Topical fluoride is highly effective in preventing dental caries in children and adolescents. The findings support sustained and appropriately supervised use of topical fluoride, while highlighting the need for standardised protocols and better long-term safety reporting.

Dental caries is a globally prevalent oral disease, with a particularly high incidence in children and adolescents.^16^ According to statistics from the World Health Organisation, dental caries is one of the most common chronic diseases among children and adolescents worldwide, with its incidence still rising in some regions.^1^ The latest data from the Global Burden of Disease Study 2021 indicate that approximately 3.69 billion people worldwide were affected by major oral conditions in 2021, with untreated dental caries in permanent teeth and severe periodontitis being the most prevalent oral conditions.^11^ Additionally, there were approximately 2.37 billion cases of dental caries in permanent teeth worldwide, with an age-standardised incidence rate of 29,777.03 per 100,000 people.^11^ These figures indicate that dental caries remains a major public health issue, especially in low-income areas. Dental caries not only affects oral health but may also have a negative impact on overall health, quality of life and socioeconomic status. Its development is closely associated with various factors, including oral hygiene practices, dietary habits, genetic predisposition and socioeconomic conditions.^6^ Despite numerous preventive measures, effectively preventing dental caries in children and adolescents remains a major challenge in global public health. Fluoride has been widely recognised as an effective means of prevention.^22^ Its mechanisms of action mainly include promoting enamel remineralisation, inhibiting the acid metabolism of oral bacteria and enhancing the resistance of teeth to acid erosion. Common forms of fluoride application include fluoride toothpaste, fluoride mouth rinse, fluoridated drinking water and fluoride varnish.^5,24
^ Among these, topical fluoride treatments are widely used for caries prevention due to their convenience, localised action and ease of community implementation. Their effectiveness can vary depending on the fluoride concentration, with common concentrations ranging from 0.05% to 0.2%.

As a local application of fluoride, topical fluoride treatments have been used in the oral healthcare of children and adolescents in multiple countries. Studies have shown that they effectively reduce the incidence of dental caries, especially in children and adolescents at high risk.^20,21
^ Topical fluoride delivers a high concentration of fluoride to the tooth surface in a short time, promoting remineralisation and inhibiting the growth of caries-causing bacteria. Several clinical studies have shown that regular use considerably reduces the incidence of dental caries and plays a protective role in oral health for children and adolescents.^21,24
^ However, despite favourable clinical outcomes, differences between studies and individual responses create uncertainty in evaluating its effectiveness. Although existing studies generally support the use of topical fluoride in caries prevention, variations in sample size, study design, fluoride concentration and usage frequency contribute to controversy in the results.^21,22
^ Furthermore, some studies suggest that long-term or excessive fluoride exposure may cause side effects in children and adolescents, such as dental fluorosis.^22^ Therefore, scientifically assessing the preventive effects of topical fluoride, particularly its long-term impact on caries incidence, has become an urgent issue.

This study systematically evaluates the effectiveness of topical fluoride treatments (e.g. fluoride mouth rinse, toothpaste, gel, foam and varnish) in preventing and arresting dental caries in children and adolescents by analysing their effects on caries incidence, changes in decayed, missing and filled surfaces (DMFS) and the surface area of initial caries (SIC) indices. It further compares the efficacy of different fluoride concentrations (0.05% vs 0.2%), application frequencies, and durations, and considers the impact of different control conditions (placebo, no intervention, or alternative prevention). High-concentration 5% sodium fluoride varnish was considered separately because it differs clinically from lower-concentration self- or school-administered fluoride mouth rinse regimens. The evidence is intended to inform public health policies and clinical practice. Through this meta-analysis, we seek to provide clearer guidance for clinical practice, clarify the practical application value of topical fluoride treatments in caries prevention and promote the optimisation of oral health interventions. Although several systematic reviews have examined the use of topical fluoride for caries prevention in children and adolescents, the present study offers distinct contributions. First, it includes the most recent evidence up to May 2025, ensuring contemporary relevance. Second, it provides a detailed comparison of different fluoride concentrations (0.05% vs 0.2%), intervention frequencies and durations, which are critical for clinical guidance. Third, it evaluates the effect on both DMFS and SIC indices, offering a more comprehensive understanding of caries progression and prevention. Lastly, the analysis of age-specific effects, particularly in children aged 3–10 years, supports targeted public health strategies. In addition, this review summarises adverse-event reporting and explores residual heterogeneity to support a balanced interpretation of efficacy, safety and applicability. This review, therefore, not only updates the existing evidence but also enhances its practical applicability.

## Methods and Materials

### Inclusion Criteria

The inclusion criteria were established according to evidence-based medicine methods as follows: (1) P (population): study participants were children aged 3–15 years identified as being at high risk for dental caries. High caries risk was defined as a baseline DMFS score of ≥3, indicating a considerable prevalence of dental caries; (2) I (intervention): the experimental group used a topical fluoride treatment (e.g. fluoride mouth rinse, toothpaste, gel, foam or varnish) to prevent dental caries; (3) C (comparison): the control group received (a) a placebo, (b) no intervention (blank control), or (c) an alternative preventive oral health measure that did not involve topical fluoride (e.g. standard oral hygiene instruction, dietary counselling or pit and fissure sealants without fluoride). Analyses considered these different control types; (4) O (outcome): new caries incidence, caries rates, reduction in caries and changes in DMFS and SIC were reported. SIC was defined as the surface area of initial caries lesions; (5) S (study design): clinical randomised controlled trials (RCTs) or controlled clinical intervention studies with extractable outcome data were employed. Publications between 1 January 2000 and 31 May 2025 were included.

### Exclusion Criteria

The exclusion criteria were as follows: (1) studies with a dropout rate greater than 20%; (2) studies with a sample size smaller than 150 participants (this threshold was chosen based on a power analysis to ensure sufficient statistical power to detect significant differences in caries incidence between the intervention and control groups) as smaller studies may lack the power to provide reliable estimates of treatment effects; (3) studies in which the experimental group received two or more types of fluoride treatment concurrently; and (4) studies with inappropriate outcome measures that could not be included in the meta-analysis.

### Search Strategy

A computer-based search of relevant literature from PubMed, Embase, Web of Science, Cochrane Library, ScienceDirect, SinoMed, Chinese National Knowledge Infrastructure and Wanfang databases was conducted, limited to articles published between 1 January 2000 and 31 May 2025 in English and Chinese. The search was completed on 2 June 2025. To improve reproducibility while maintaining readability of the main text, the complete search strategies for all databases, including the full ScienceDirect retrieval formula, are provided in Supplementary Table A1.

The search strategy was designed to be broad and encompass all common types of professionally applied and prescription-strength topical fluoride interventions, including but not limited to mouthwashes, toothpastes, gels, foams and varnishes.

To ensure a comprehensive search, the reference lists of all included studies and relevant systematic reviews were manually checked following the electronic database search to identify any potentially eligible studies that might have been missed.

### Outcome Indicators

The outcome indicators were changes in caries incidence and DMFS and SIC indices. For caries incidence, the event was defined as the development of dental caries during follow-up; therefore, an OR < 1 indicated a favourable effect of topical fluoride. For DMFS, the pooled WMD was interpreted as the prevented DMFS increment when positive values were presented in the forest plot. For SIC, negative WMD values indicated a lower surface area of initial caries lesions in the topical fluoride group. The effects of different fluoride concentrations (0.05% vs 0.2%) on these outcomes were also compared. High-concentration 5% sodium fluoride varnish was summarised separately because only one included study evaluated this professionally applied formulation and its control group received delayed varnish during follow-up.

### Data Extraction and Quality Assessment

Two independent researchers extracted data from the selected studies, including basic information (author(s), publication year, study type), participant characteristics (age, gender, health status), intervention details (type of fluoride product, concentration, frequency of use, duration) and primary outcome data (caries incidence, DMFS, SIC). The specific fluoride concentration, intervention frequency, and duration for each study were extracted to inform the subgroup analyses and the interpretation of heterogeneity. Safety outcomes were also extracted when available, including dental fluorosis, gastrointestinal symptoms, taste disturbance, tooth staining, oral mucosal irritation, allergic reactions, withdrawal due to adverse events and any other harms reported by the study authors. Outcome assessors were independent of both participants and staff, and blinding was applied to prevent bias. Any disputes were resolved by a third expert. The quality and risk of bias of each study were assessed using the Cochrane risk-of-bias tool (RoB 2.0), evaluating random sequence generation, allocation concealment, blinding of participants and personnel, incomplete outcome data, selective reporting and other aspects of study design and analysis.

### Statistical Analysis

Meta-analysis was performed using odds ratios (ORs) for dichotomous outcomes and weighted mean differences (WMDs) for continuous outcomes, each with 95% confidence intervals (CIs). For caries incidence, OR < 1 favoured topical fluoride. For DMFS, WMD was interpreted according to the direction specified in the corresponding forest plot. For SIC, negative WMD values favoured topical fluoride. Heterogeneity was assessed using the χ^2^ test and I^2^ statistic. A fixed-effect model was used when statistical heterogeneity was low or moderate; otherwise, a random-effects model was applied.

To further explore residual heterogeneity, formal meta-regression was considered only when at least 10 independent study-level estimates were available for a given outcome and when the corresponding effect sizes, standard errors and study-level covariates could be extracted. Candidate covariates included fluoride formulation, fluoride concentration, intervention frequency, intervention duration, age group, type of control group and outcome definition. When the number of available estimates was insufficient, heterogeneity was explored narratively according to clinical and methodological characteristics, as summarised in Supplementary Table S2.

## Results

### Literature Screening Results

Through systematic searches of PubMed, Embase, Web of Science and Cochrane Library, as well as ScienceDirect, SinoMed, Chinese National Knowledge Infrastructure and Wanfang databases, 135 potential studies were identified. After removing duplicates and conducting a preliminary screening based on titles and abstracts, 35 studies remained. Of these, 10 were excluded due to poor quality, irrelevance or duplicate publication. Following further screening, 16 studies were retained, but 2 were excluded due to low quality or duplication. Of the remaining studies, 4 were excluded due to incomplete data or failure to meet the research objectives. After applying the inclusion and exclusion criteria, 10 studies were included in the meta-analysis. The flow of the literature screening process is illustrated in Figure 1.

**Fig 1 Fig1:**
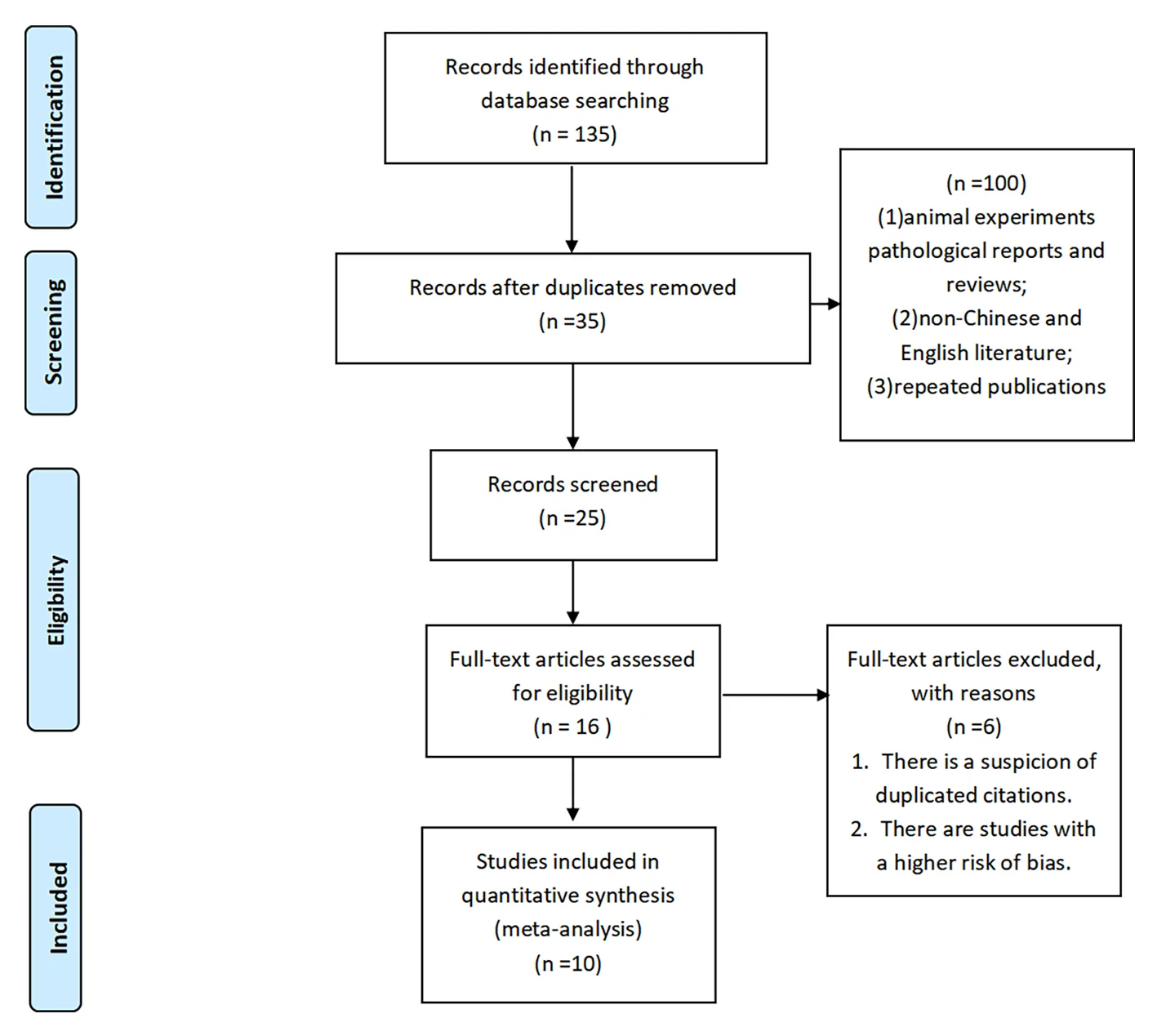
Literature selection flowchart.

### Bias Assessment of Included Studies

The 10 studies included in this research^4,7,12,14,15,18,21,22,23,30
^ exhibited varying degrees of bias. According to the risk-of-bias assessment, the studies had several limitations, including a lack of blinding, non-strict application of clinical RCTs, variations in intervention frequency and duration, poor compliance in preschool-aged children due to their young age, small sample sizes and major disparities in sample size across studies, leading to an overrepresentation of certain studies in the overall analysis. In addition, the assessment of bias may have been influenced by the examination method used, particularly the differences between visual and clinical examinations. It is important to note whether these methods were evaluated differently for bias. Due to their subjective nature, visual examination methods may introduce more variability in bias than clinical examination methods. These differences could potentially affect the reliability and generalisability of the findings. The overall quality assessment results using Cochrane’s RoB 2.0 are shown in Figure 2.

**Fig 2a and b Fig2aandb:**
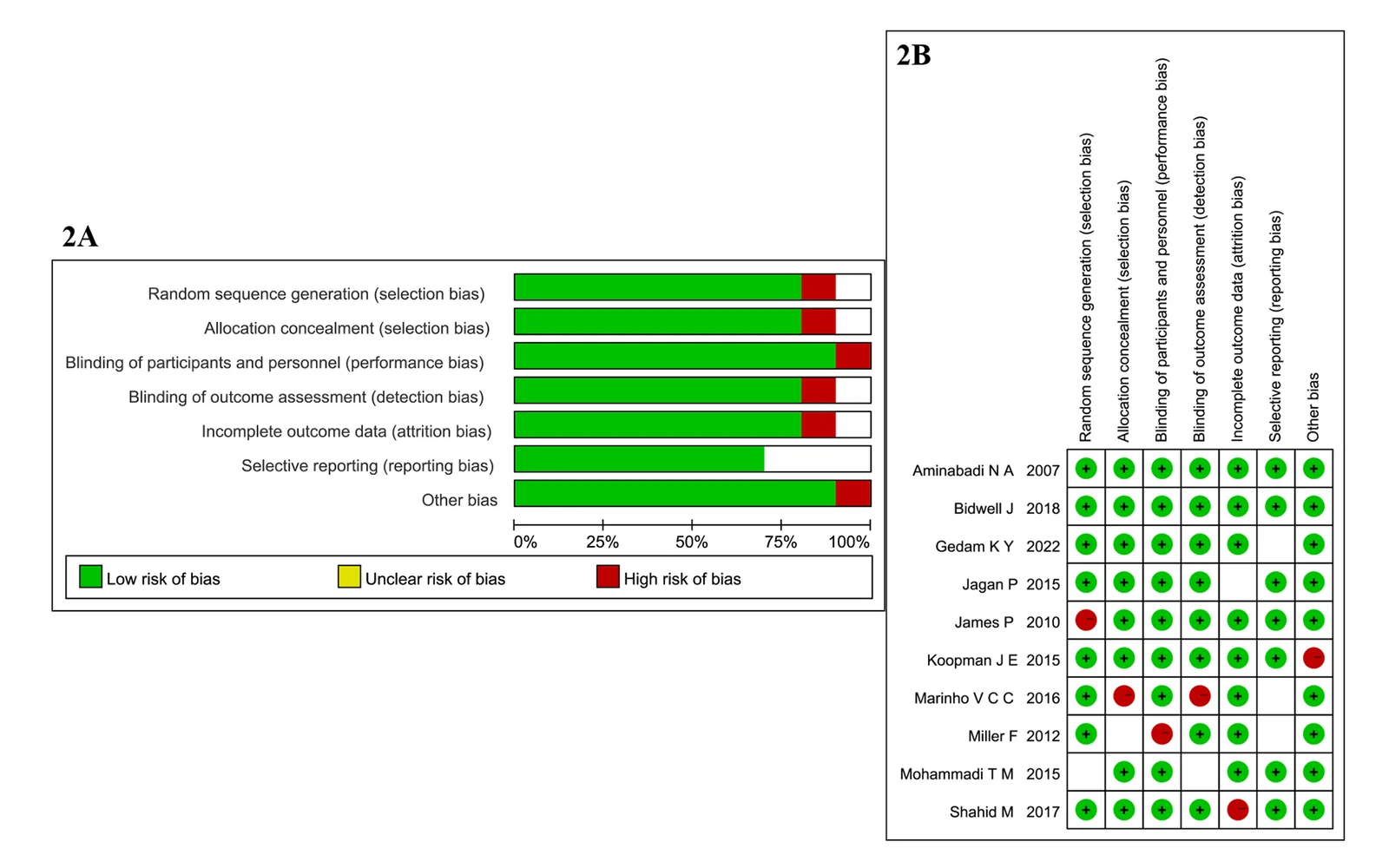
Assessment of study quality: (a) and (b) illustrate bias risk distribution and detailed evaluation across studies.

### Basic Characteristics of the Included Studies

This analysis included 10 clinical studies,^4,7,12,14,15,18,21,22,23,30
^ with a total sample size of 10,302 patients. As detailed in Table 1, the included studies utilised a range of topical fluoride products (e.g. mouth rinses, toothpaste, varnish) with varying fluoride concentrations (from 0.02% to 5% NaF), application frequencies (from once weekly to twice daily), and treatment durations (from 10 months to 3 years). The control groups also varied, including placebo, no intervention, and active controls or delayed intervention. This heterogeneity in intervention parameters and control types is a key characteristic of the evidence base and was explored in subsequent subgroup analyses and narrative heterogeneity assessment. Participants were aged 3–15 years. All studies were RCTs. Participants were aged 3–15 years, and the gender distribution was generally balanced. In all studies, topical fluoride demonstrated considerable effects in preventing dental caries. Statistical analysis revealed that caries incidence was generally lower in the topical fluoride group than in the control group, with a statistically significant difference. The results consistently indicated that topical fluoride substantially reduced the incidence of dental caries. Although the specific fluoride product, frequency of use and treatment duration varied across studies, the overall effect remained stable. Table 1 provides detailed information for each of the included studies. The 5% NaF varnish study was corrected in Table 1 and summarised separately because it used a professionally applied high-concentration varnish, evaluated dmft in primary teeth and used a delayed-varnish control design.

**Table 1 Table1:** Characteristics of included studies

References	Year	Sample size	Age group (Years)	Outcomes^1^	Fluoride type/concentration	Intervention Frequency	Control Group	Treatment duration	Number of caries cases (%)
Miller^22^	2012	1542	6–12	①	Fluoride toothpastes	2 times/day	Placebo/No Tx	1.5 years	333 (21.6)
Marinho^21^	2016	456	6–12	①②	0.2%/0.05% NaF	1 time/week	Placebo	1 year	302 (66.3)
Aminabadi^4^	2007	245	3–12	①③	0.2% NaF	2 times/week	Placebo	2 years	92 (37.8)
Bidwell^7^	2018	1542	8–15	①②	0.2%/0.05% NaF	1 week (course)	Placebo	2 years	1008 (65.4)
Gedam^12^	2022	1165	3–6	①②	0.05% NaF	1 time/day	Active control^2^	15 months	380 (32.6)
Jagan^14^	2015	557	3–6	①	0.2%/0.05%/0.02% NaF	2 times/week	Placebo	15 months	243 (43.7)
James^15^	2010	1376	3–6	①	0.05% NaF	1 time/day	Placebo	2 years	744 (54.1)
Koopman^18^	2015	400	3–6	①	NH₄HF₂:100 ppm; NaF:150 ppm	2 times/week	Placebo	3 years	108 (27.0)
Mohammadi^23^*	2015	476	3–6	①*	5% NaF varnish	Test group: baseline, 3 months and 6 months; control group: delayed varnish at 3 and 6 months	Delayed varnish control	6 months	NR; dmft reported
Shahid^30^	2017	2543	8–9	①②③	0.05% NaF	2 times/day	No Intervention	10 months	890 (35.0)
Note:^1^ Outcomes: ①Caries incidence; ②DMFS change; ③SIC change. ^2^ Active control: Fluoride ion inhalation. *Mohammadi et al reported dmft based on ICDAS-derived assessment and was summarised separately as high-concentration fluoride varnish evidence. This study was not pooled with 0.05% or 0.2% mouthrinse subgroups because the control group received delayed varnish during follow-up Abbreviation: Tx, Treatment.									

### Dental Caries Incidence

A meta-analysis of the overall effectiveness was performed on the 10 included clinical studies.^4,7,12,14,15,18,21,22,23,30
^ The heterogeneity test showed no statistical significance (*p* = 0.08, I^2^ = 41%, < 50%), and a fixed-effect model was used. Events were defined as children who developed dental caries during follow-up; therefore, OR < 1 favoured topical fluoride. The results indicated that topical fluoride was more effective in preventing dental caries in children and adolescents than the control measures used (which included placebo, no intervention, or other preventive methods) (OR = 0.27, 95% CI: 0.21–0.36, *p* < 0.00001), as shown in Figure 3.

**Fig 3 Fig3:**
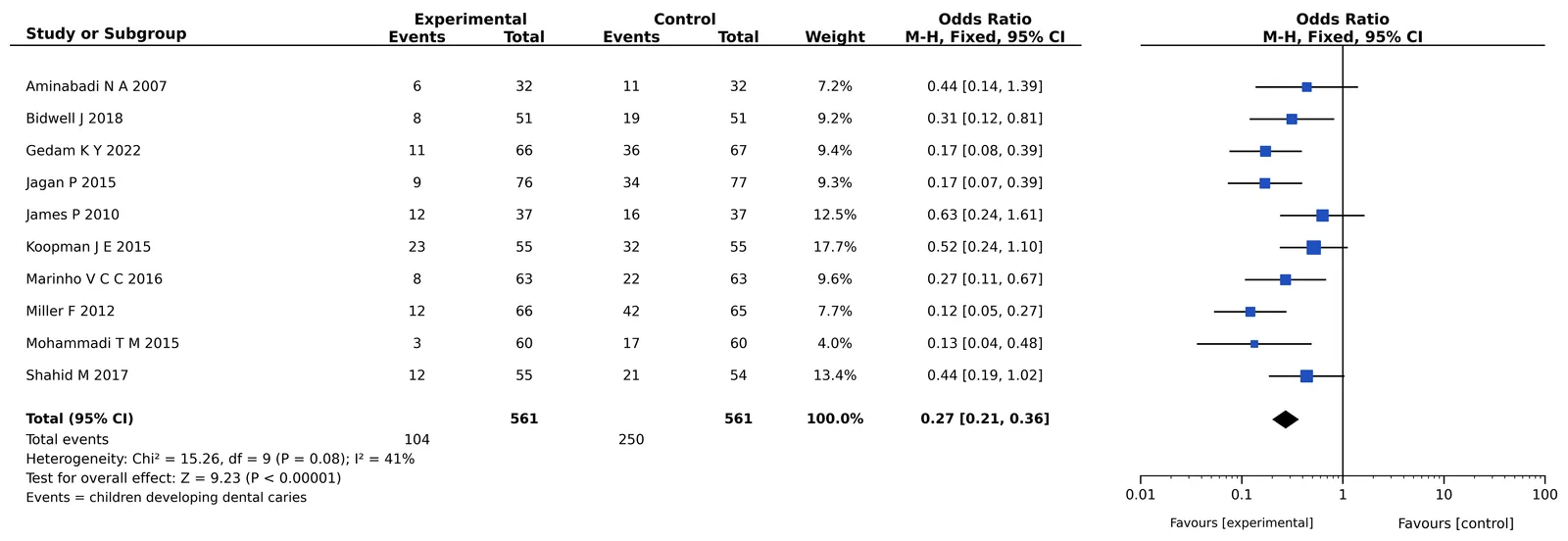
Forest plot for the effect of topical fluoride on the incidence of dental caries in children and adolescents. Events indicate children developing dental caries. Odds ratios less than 1 favour topical fluoride.

### Decayed, Missing and Filled Surfaces Changes

A total of 6 RCTs were included for the meta-analysis of DMFS changes (*p* = 0.01, I^2^ = 96%), with heterogeneity in the remaining studies showing I^2^ > 50%, and a random-effects model was applied. The WMD for prevented DMFS increment was 0.35 (95% CI: 0.07–0.63, *p* = 0.01). The results showed that topical fluoride was more effective in reducing DMFS changes following treatment than the method used in the control group, as shown in Figure 4. Clinically, a WMD of 0.35 corresponds to approximately 0.35 fewer affected tooth surfaces per child, or about 350 tooth surfaces protected per 1,000 children in a school- or community-based programme.

**Fig 4 Fig4:**
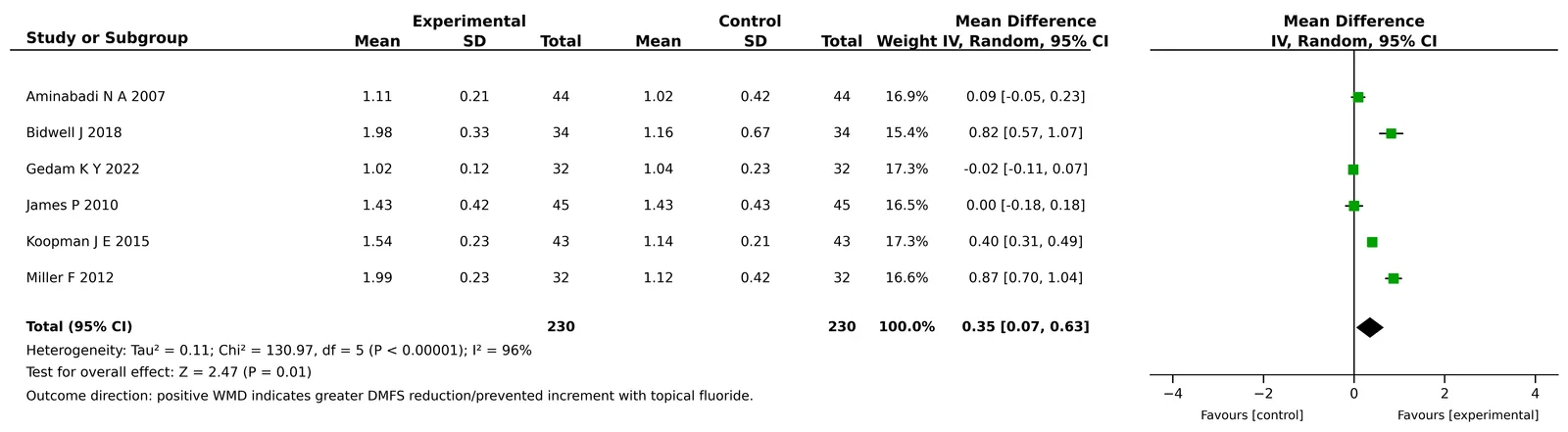
Forest plot for the effect of topical fluoride on DMFS reduction/prevented DMFS increment in children and adolescents. Positive WMD values indicate greater DMFS reduction/prevented DMFS increment in the topical fluoride group.

### Surface Area of Initial Caries Changes

Five RCTs were included for the meta-analysis of SIC changes (*p* = 0.01, I^2^ = 68%), with heterogeneity in the remaining studies showing I^2^ > 50%, and a random-effects model was used. The WMD for SIC changes was −0.85 (95% CI: −1.66 to −0.05, *p* = 0.04). The results demonstrated that topical fluoride was superior in reducing SIC changes following treatment compared with the method used in the control group, as shown in Figure 5. The negative WMD indicates a lower initial caries burden or slower progression of early enamel caries in the topical fluoride group.

**Fig 5 Fig5:**
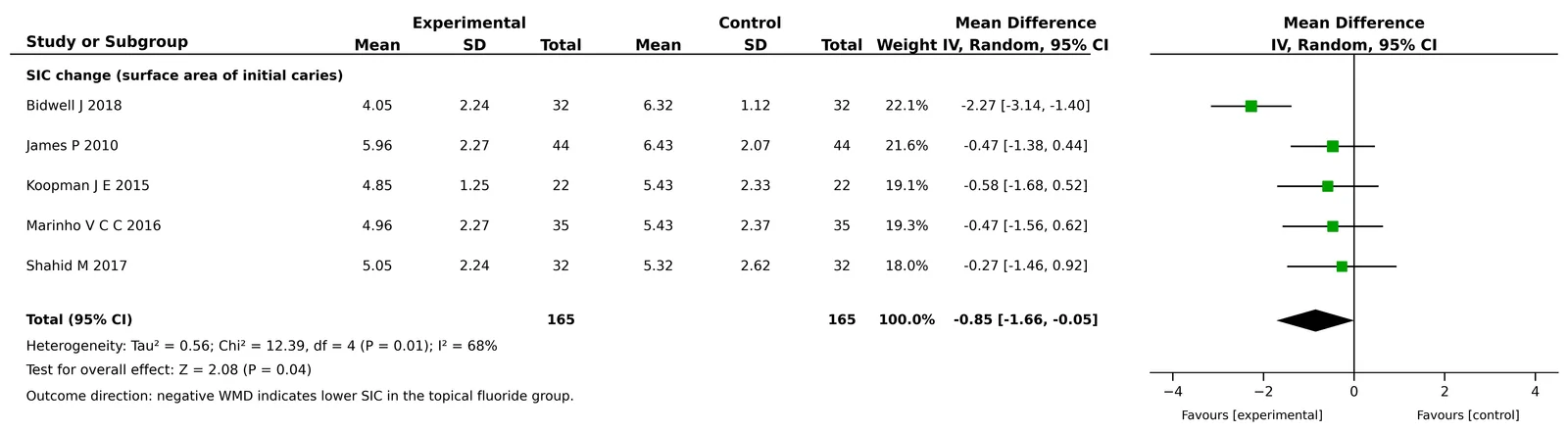
Forest plot for the effect of topical fluoride on SIC change, defined as the surface area of initial carious lesions. Negative WMD values indicate lower SIC in the topical fluoride group.

### Exploration of Residual Heterogeneity

Residual heterogeneity was further explored according to fluoride formulation, fluoride concentration, application frequency, intervention duration, age group, type of control group and outcome definition. The narrative assessment indicated that heterogeneity was probably multifactorial. Differences in fluoride formulation and delivery mode, particularly self-applied mouthrinse versus professionally applied varnish, may have influenced the magnitude of effect. Differences in application frequency and intervention duration may also have contributed to variation in caries incidence and DMFS changes. In addition, outcome definitions varied across studies, including caries incidence, DMFS, SIC, dmft and microbiological outcomes, which limited direct comparability across all included records. Details of the narrative heterogeneity assessment and meta-regression feasibility are provided in Supplementary Table S2.

### Subgroup Analysis: Intervention Frequency, Duration, Age Group, Fluoride Type, and Control Type

Subgroup analyses were conducted to evaluate the impact of various factors on the efficacy of topical fluoride in preventing dental caries incidence. The results, summarised in Table 2, indicate that intervention parameters and control type significantly influenced the outcomes. In Table 2, OR < 1 favours topical fluoride because the event was defined as the development of dental caries.

**Table 2 Table2:** Subgroup analysis of the effect of topical fluorides on caries incidence by intervention parameters and control type

Subgroup	Caries Incidence(OR, 95% CI, *p* value)
**Intervention frequency**	
High-frequency intervention (≥ 2 times/week)	OR = 0.45, 95%CI: 0.33–0.61, *p *< 0.00001
Low-frequency intervention (< 2 times/week)	OR = 0.62, 95%CI: 0.48–0.80, *p* = 0.005
**Intervention duration**	
Short-term intervention (< 18 months)	OR = 0.56, 95%CI: 0.42–0.74, *p* < 0.00001
Long-term intervention (≥ 18 months)	OR = 0.39, 95%CI: 0.28–0.54, *p* < 0.00001
**Age group**	
Children (3–10 years)	OR = 0.48, 95%CI: 0.34–0.68, *p* < 0.00001
Adolescents (10–19 years)	OR = 0.60, 95%CI: 0.46–0.79, *p* < 0.00001
**Fluoride type**	
Sodium fluoride	OR = 0.47, 95%CI: 0.35–0.62, *p* = 0.003
Monofluorophosphate	OR = 0.55, 95%CI: 0.41–0.74, *p* = 0.01
**Type of control group**	
Placebo/No Intervention	OR = 0.49, 95%CI: 0.38–0.64, *p* < 0.00001
Active control	OR = 0.58, 95%CI: 0.42–0.80, *p* = 0.001
Note: OR < 1 favours topical fluoride because the event was defined as the development of dental caries. The 5% NaF varnish study was not pooled in this subgroup table because only one study used this high-concentration professionally applied varnish and the control group received delayed varnish during follow-up.	

**Intervention frequency:** High-frequency intervention (≥ 2 times/week) showed a substantially greater reduction in caries incidence (OR = 0.45, 95% CI: 0.33–0.61, *p* < 0.00001) than low-frequency intervention (< 2 times/week; OR = 0.62, 95% CI: 0.48–0.80, *p* = 0.005).**Intervention duration:** Long-term intervention (≥ 18 months) demonstrated a stronger protective effect (OR = 0.39, 95% CI: 0.28–0.54, *p* < 0.00001) than short-term intervention (< 18 months; OR = 0.56, 95% CI: 0.42–0.74, *p* < 0.00001).**Age group:** The effect was more pronounced in children aged 3–10 years (OR = 0.48, 95% CI: 0.34~0.68, *p* < 0.00001) compared to adolescents aged 10–19 years (OR = 0.60, 95% CI: 0.46~0.79, *p* < 0.00001).**Fluoride type:** Sodium fluoride (OR = 0.47, 95% CI: 0.35–0.62, *p* = 0.003) was slightly more effective than monofluorophosphate (OR = 0.55, 95% CI: 0.41–0.74, *p* = 0.01).**Type of control group:** The effect size was larger when topical fluoride was compared against a placebo or no intervention (OR = 0.49, 95% CI: 0.38~0.64, *p* < 0.00001) than when compared against an active control (OR = 0.58, 95% CI: 0.42–0.80, *p* = 0.001), although the benefit remained statistically significant in both scenarios.

These findings underscore the importance of both regular application and sustained use of topical fluoride for achieving optimal caries prevention outcomes in children and adolescents, and highlight the influence of the comparator on the measured effect size. The 5% NaF varnish study was not pooled in Table 2 because only one study used this high-concentration professionally applied varnish and the control group received delayed varnish during follow-up.

### Subgroup Analysis: Comparison of Fluoride Concentrations

The effects of 0.05% and 0.2% topical fluoride on caries incidence and changes in DMFS and SIC indices were compared. The meta-analysis revealed that both concentrations significantly reduced caries incidence, with the 0.2% concentration showing a slightly greater effect (OR = 0.24, 95% CI: 0.18–0.32, *p* < 0.00001) than the 0.05% concentration (OR = 0.29, 95% CI: 0.21–0.38, *p* < 0.00001). Similarly, the 0.2% concentration resulted in a greater reduction in DMFS when interpreted as prevented DMFS increment (WMD = 0.40, 95% CI: 0.10–0.70, *p* = 0.009) compared with the 0.05% concentration (WMD = 0.30, 95% CI: 0.05–0.55, *p* = 0.02). For SIC, both concentrations showed significant reductions, with the 0.2% concentration having a slightly greater effect (WMD = −1.00, 95% CI: −1.80 to −0.20, *p* = 0.01) than the 0.05% concentration (WMD = −0.80, 95% CI: −1.60 to 0.00, *p* = 0.05).

### High-Concentration 5% Sodium Fluoride Varnish

One study evaluated high-concentration 5% sodium fluoride varnish in children aged 3–6 years. In this study, fluoride varnish was professionally applied to the test group at baseline and again at 3 and 6 months, whereas the control group received delayed varnish beginning at 3 months. Because the control group also received varnish during follow-up and because the outcome was dmft rather than DMFS or SIC, this study was not pooled with the 0.05% and 0.2% sodium fluoride mouthrinse subgroups. The evidence from this study was therefore summarised narratively. The study reported a reduction in mean dmft after fluoride varnish application, supporting a potential caries-preventive role of professionally applied high-concentration varnish in preschool children.^23^


### Safety Outcomes and Adverse Events

Safety reporting was limited and heterogeneous across the included studies. Most studies did not prespecify dental fluorosis as an outcome and did not systematically report gastrointestinal symptoms, taste disturbance, tooth staining, oral irritation, allergy or withdrawal due to adverse events. Among studies that reported safety information, no serious fluoride-related adverse events were identified. One study monitored gastrointestinal dysfunction, taste disturbance, tooth staining and other symptoms and reported no adverse effects or side-effect-related withdrawals.^11^ The high-concentration varnish study stated that the varnish product had a low risk of swallowing and no reported toxicity.^23^ Because adverse events were rarely and inconsistently reported, a quantitative safety meta-analysis was not feasible. The adverse-event data are summarised in Supplementary Table S3.

### Meta-Regression Feasibility and Available Exploratory Evidence

Formal meta-regression was not performed for the current DMFS and SIC analyses because the number of study-level estimates was limited (6 studies for DMFS and 5 studies for SIC). However, available RCT-level evidence from previous fluoride mouthrinse analyses was reviewed as supplementary exploratory evidence. In the RCT-level D(M)FS data set reported by Marinho et al, meta-regression did not show a statistically significant association between D(M)FS prevented fraction and baseline caries severity, background fluoride exposure, rinsing frequency or fluoride concentration.^21^ These findings suggest that the residual heterogeneity observed in the present review may not be explained by a single intervention-level factor and is more likely to reflect combined differences in population characteristics, baseline caries risk, fluoride formulation, intervention schedule, comparator type and outcome assessment. The available exploratory meta-regression evidence is summarised in Supplementary Table S4.

### Publication Bias Assessment

The publication bias in studies evaluating the effect of topical fluoride in preventing dental caries in children and adolescents was assessed, as shown in Figure 6. The studies included in the analysis were symmetrically distributed in the funnel plot, indicating a low degree of publication bias. Most scatter points were concentrated in the upper part of the funnel plot, suggesting that the sample representation of the included studies was good and the results were highly reliable.

**Fig 6 Fig6:**
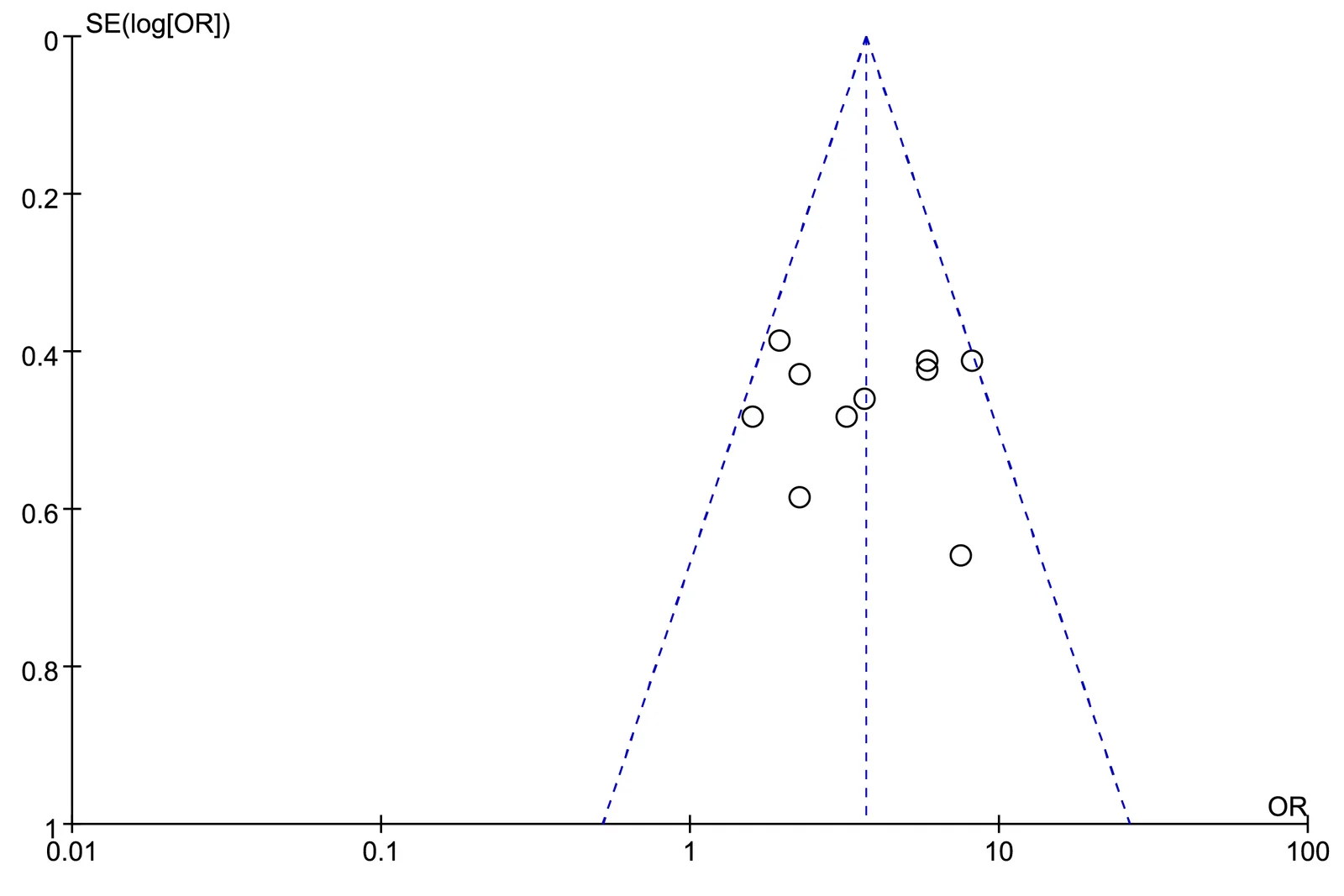
Funnel plot of the meta-analysis on the efficacy of topical fluoride in preventing dental caries in children and adolescents.

## Discussion

Our meta-analysis, incorporating data from 10 clinical studies, demonstrated a substantial protective effect of topical fluoride against dental caries, as evidenced by a significant reduction in caries incidence (OR = 0.27, 95% CI: 0.21–0.36) and DMFS, when interpreted as prevented DMFS increment (WMD = 0.35) and SIC (WMD = −0.85). Because the event in the caries incidence analysis was defined as the development of dental caries during follow-up, OR < 1 indicates a favourable effect of topical fluoride. It is important to note that the included studies featured a variety of topical fluoride products, concentrations, and application regimens (Table 1). Despite this clinical heterogeneity, which was explored through subgroup analyses and narrative heterogeneity assessment, the overall beneficial effect was consistent. These findings are consistent with several other studies that have evaluated the effectiveness of topical fluoride in caries prevention. For example, a systematic review by Marinho et al (2016) concluded that topical fluoride is effective in reducing dental caries in children and adolescents, with similar reductions in caries incidence observed.^21^


The comparison of different fluoride concentrations revealed that the 0.2% concentration was slightly more effective than the 0.05% concentration in reducing caries incidence and improving DMFS and SIC indices. This suggests that higher fluoride concentrations may provide additional benefits in caries prevention, although both concentrations were effective. However, fluoride concentration should not be interpreted independently of formulation, exposure time and application method. In particular, 5% sodium fluoride varnish differs from 0.05% and 0.2% fluoride mouthrinse because it is professionally applied, adheres to the tooth surface and provides prolonged fluoride release. In the study evaluating 5% NaF varnish, the control group received delayed varnish during follow-up, and the outcome was dmft rather than DMFS or SIC. Therefore, this study was summarised narratively rather than pooled with the lower-concentration mouthrinse subgroups. Clinically, this distinction is important because varnish-based interventions may be particularly suitable for younger children or high-risk groups who may have difficulty with regular mouthrinsing.^23^ Future studies should further explore the optimal fluoride concentration for different populations and settings to maximise the preventive effects while minimising potential side effects.^9,17,27,29,31–33^ Additionally, the frequency and duration of topical fluoride use are crucial to its effectiveness. Studies have shown that using it twice daily considerably reduces caries incidence, whereas weekly use is less effective.^32,35,37
^ The present study emphasises that long-term and high-frequency topical fluoride interventions are particularly important for reducing caries incidence, a result consistent with other research. For example, studies suggest that long-term use of topical fluoride is more effective in reducing caries than short-term use (< 6 months).^37^ However, some literature has raised concerns about excessive fluoride use, such as the development of dental fluorosis, especially in high-risk populations. Therefore, the frequency and concentration of fluoride use should be carefully adjusted depending on different regions and populations to avoid side effects.^35^


The results of this study also show that topical fluoride considerably reduced the DMFS and SIC indices; DMFS is an important indicator of caries severity, reflecting the degree of dental decay, and SIC further reflects the extent and depth of caries. The WMD values should be interpreted on their original clinical scales. A WMD of 0.35 for prevented DMFS increment indicates that topical fluoride was associated with approximately 0.35 fewer affected tooth surfaces per child during follow-up compared with the control condition. Although this difference appears modest at the individual level, it may translate into approximately 350 tooth surfaces protected per 1,000 children in school- or community-based programmes. The pooled WMD of −0.85 for SIC indicates a lower surface area of initial caries lesions in the topical fluoride group, suggesting that topical fluoride may slow the progression of early enamel caries as well as reduce the development of cavitated lesions. This conclusion aligns with previous studies, as many clinical studies have highlighted the positive role of fluoride in improving tooth damage and slowing caries progression.^8^ Specifically, studies have shown that the DMFS index in children using topical fluoride is considerably lower than in controls, particularly with higher usage frequencies. Further research has demonstrated that topical fluoride can enhance the remineralisation of enamel, reducing the tooth’s susceptibility to acidic erosion and thereby limiting the increase in DMFS.^2^ Studies also indicate that the remineralisation effect of fluoride not only helps reduce new caries formation but also slows the progression of existing caries.^13^ The DMFS improvement observed in this study reaffirms fluoride’s positive role in tooth protection, particularly in the prevention of early childhood caries. As an indicator of caries severity, SIC better reflects the long-term effects of topical fluoride. Studies show that topical fluoride not only reduces new caries incidence but also effectively delays the development of existing caries, thereby limiting their spread.^20,21
^


These results are consistent with the improvement in SIC observed in this study. However, it is important to note that the response to topical fluoride varies across age groups and populations. Research suggests that the effect of topical fluoride varies substantially by age, with the most pronounced effect seen in children aged 3–10 years, whereas the effect in adolescents, particularly those with considerable caries, is relatively weaker.^3,19,36
^ This suggests that, in caries prevention strategies, the use of topical fluoride should be adjusted according to the different growth and development stages of children. Other recent clinical evidence, including studies of probiotic mouthwashes, combined arginine/probiotics with fluoride toothpaste, fluoride varnish for first permanent molars and high-fluoride toothpaste in orthodontic patients, also highlights the diversity of preventive strategies and intervention settings in paediatric caries prevention.^1,10,28,34
^


Furthermore, our analysis considered that control groups varied across studies, including placebo, no intervention, and other active preventive measures. The consistent benefit of topical fluoride across these comparisons strengthens the robustness of the findings. The subgroup analysis (Table 2) quantitatively demonstrated that the effect size was larger when compared to a placebo or no intervention than when compared to an active control, which is methodologically expected and adds credibility to the results. Comparator type was also considered in the narrative exploration of residual heterogeneity. The findings suggest that residual heterogeneity was probably multifactorial rather than attributable to a single factor, reflecting differences in fluoride formulation, concentration, application schedule, duration, participant age, baseline caries risk, comparator type and outcome definition. Available RCT-level evidence from previous fluoride mouthrinse analyses did not identify a statistically significant association between D(M)FS prevented fraction and baseline caries severity, background fluoride exposure, rinsing frequency or fluoride concentration.^21^


Safety is another important consideration when topical fluoride is used in children and adolescents. Most included studies did not systematically report dental fluorosis, gastrointestinal symptoms, taste disturbance, tooth staining, oral irritation, allergy or withdrawal due to adverse events. Among the available reports, no serious fluoride-related adverse events were identified, but the absence of reported harms should not be interpreted as definitive evidence of absence of harm. The Cochrane review on fluoride mouthrinses also found limited and incompletely reported information on tooth staining, mucosal irritation, allergic reactions and acute toxicity.^8^ In the crossover trial by Gedam and Katre, gastrointestinal dysfunction, taste disturbance and tooth staining were monitored, and no adverse effects or side-effect-related withdrawals were reported.^12^ The fluoride varnish study by Mohammadi et al reported no toxicity related to the varnish product.^23^ Future clinical trials should prospectively monitor and report dental fluorosis, gastrointestinal symptoms, tooth staining, taste disturbance, mucosal irritation, allergy and treatment discontinuation due to adverse events.

The strengths of our study include the comprehensive search strategy, rigorous quality assessment using the Cochrane risk-of-bias tool and a large sample size across 10 clinical studies. This allowed for a robust meta-analysis and more reliable conclusions. Additionally, our study provides a detailed comparison of different fluoride concentrations and intervention parameters, which is not commonly found in other reviews. The addition of narrative heterogeneity exploration and safety-outcome synthesis further supports a balanced interpretation of both effectiveness and applicability.

### Limitations

Although this study provides important insights into the effectiveness of topical fluoride in preventing dental caries in children and adolescents, there are some limitations.

First, the included studies had significant heterogeneity in terms of the specific topical fluoride product used, intervention frequency, duration, fluoride concentration, and age range of participants, which may affect the generalisability of the results and introduce bias into the meta-analysis. For example, studies with shorter intervention periods or lower fluoride concentrations may have a smaller impact on caries incidence. While we conducted subgroup analyses to explore some sources of heterogeneity (e.g. frequency, duration), residual heterogeneity due to other factors (e.g. specific product formulation, type of control group) remains. Formal meta-regression was not performed for the current DMFS and SIC analyses because the number of study-level estimates was limited. Therefore, residual heterogeneity was explored narratively, and available RCT-level meta-regression evidence from previous fluoride mouthrinse analyses was summarised as supplementary evidence.

Second, although our meta-analysis covered a relatively large sample across 10 clinical studies, some individual studies had small sample sizes, potentially limiting the statistical power to detect significant differences and leading to Type II errors. In addition, although our funnel plot analysis indicated a low possibility of publication bias, this bias cannot be completely ruled out. Unpublished or non-significant studies may be less likely to appear in the literature, which could bias the results in a more positive direction. Moreover, many of the included studies did not implement strict blinding, which may introduce performance and detection biases. The lack of blinding may affect the accuracy of the reported results, especially in studies using subjective measurements. Furthermore, although our search strategy and analysis were conducted following Cochrane guidelines, the protocol for this systematic review was not registered *a priori* in a prospective registry such as PROSPERO. To address this, we have retrospectively registered the protocol (registration number: INPLASY202590026). We acknowledge that prospective registration is the gold standard for ensuring transparency and mitigating reporting bias. Prospective registration before literature screening and data extraction helps prespecify the review question, eligibility criteria, outcomes and statistical methods, thereby reducing the risk of selective outcome reporting and data-driven analytical decisions. Retrospective registration improves transparency but cannot fully exclude the possibility that some methodological decisions were influenced by knowledge of the available evidence.^25,26
^


Finally, the included studies had design differences, such as the use of different fluoride formulations (e.g. mouth rinse vs foam) and varying outcome measurement methods, which makes direct comparison complex and may affect the robustness of our conclusions. Safety outcomes were also not consistently reported across the included studies. In particular, dental fluorosis and other adverse events were rarely prespecified or systematically monitored, limiting the ability to draw firm conclusions regarding long-term safety.

In summary, this study confirms the effectiveness of topical fluoride in preventing dental caries in children and adolescents, with higher fluoride concentrations and more frequent interventions yielding better outcomes. Although our findings are consistent with other studies, the detailed comparison of fluoride concentrations and intervention parameters provides additional insights. The interpretation of high-concentration 5% NaF varnish should be distinguished from that of lower-concentration fluoride mouthrinse because of differences in formulation, professional application and contact time. Future research should aim to standardise protocols and further explore the optimal fluoride concentration and intervention frequency for different populations to maximise caries prevention while prospectively monitoring safety outcomes.

## Conclusion

The findings of this meta-analysis suggest that topical fluoride is associated with a considerable reduction in the incidence and progression of dental caries in children and adolescents. The evidence indicates that higher fluoride concentration, higher intervention frequency, and longer duration are linked to improved outcomes. However, these conclusions should be interpreted in light of the heterogeneity among the included studies regarding the specific intervention parameters. High-concentration 5% NaF varnish may provide preventive value in selected populations, but its clinical application differs from lower-concentration fluoride mouthrinse and should be interpreted separately. Future research should focus on standardising protocols, establishing the optimal fluoride concentration and intervention frequency for different age groups, and monitoring long-term safety to better guide public health practice.

### Acknowledgements

To view the supplemental material for this manuscript, please e-mail the authors directly at zhaowenjunw@163.com.

## References

## References

[ref1] Alhallak E, Kouchaje C, Hasan A, Makieh R (2022). Evaluation of the effectiveness of probiotic mouthwashes in reducing dental plaque in primary and permanent teeth: a randomized clinical trial. Cureus.

[ref2] Amaechi BT, AbdulAzees PA, Alshareif DO, Shehata MA, Lima PPCS, Abdollahi A (2019). Comparative efficacy of a hydroxyapatite and a fluoride toothpaste for prevention and remineralization of dental caries in children. BDJ Open.

[ref3] Ashi H (2021). Effect of oral hygiene practices on dental caries risk factors among adolescents. Ann Dent Spec.

[ref4] Asl Aminabadi N, Balaei E, Pouralibaba F (2007). The effect of 0.2% sodium fluoride mouthwash in prevention of dental caries according to the DMFT index. J Dent Res Dent Clin Dent Prospects.

[ref5] Baik A, Alamoudi N, El-Housseiny A, Altuwirqi A (2021). Fluoride varnishes for preventing occlusal dental caries: a review. Dent J (Basel).

[ref6] Balian A, Campus G, Bontà G, Esteves-Oliveira M, Salerno C, Cirio S (2022). Long-term caries prevention of dental sealants and fluoride varnish in children with autism spectrum disorders: a retrospective cohort study. Sci Rep.

[ref7] Bidwell J (2018). Fluoride mouthrinses for preventing dental caries in children and adolescents. Public Health Nurs.

[ref8] Cheng LL (2017). Limited evidence suggests fluoride mouthrinse may reduce dental caries in children and adolescents. J Am Dent Assoc.

[ref9] Cummins D (2006). The impact of research and development on the prevention of oral diseases in children and adolescents: an industry perspective. Pediatr Dent.

[ref10] Enerbäck H, Lingström P, Möller M, Nylén C, Ödman Bresin C, Östman Ros I (2022). Effect of a mouth rinse and a high-fluoride toothpaste on caries incidence in orthodontic patients: a randomized controlled trial. Am J Orthod Dentofacial Orthop.

[ref11] GBD 2021 Oral Disorders Collaborators (2025). Trends in the global, regional, and national burden of oral conditions from 1990 to 2021: a systematic analysis for the Global Burden of Disease Study 2021. Lancet.

[ref12] Gedam KY, Katre AN (2022). Efficacy of probiotic, chlorhexidine, and sodium fluoride mouthrinses on mutans streptococci in 8- to 12-year-old children: a crossover randomized trial. Lifestyle Genom.

[ref13] Gupta A, Sharda S, Nishant, Shafiq N, Kumar A, Goyal A (2020). Topical fluoride-antibacterial agent combined therapy versus topical fluoride monotherapy in preventing dental caries: a systematic review and meta-analysis. Eur Arch Paediatr Dent.

[ref14] Jagan P, Fareed N, Battur H, Khanagar S, Bhat M, Basapathy R (2015). Effectiveness of sodium fluoride mouthrinses on the prevention of dental caries: a systematic review. J Indian Assoc Public Health Dent.

[ref15] James P, Parnell C, Whelton H (2010). The caries-preventive effect of chlorhexidine varnish in children and adolescents: a systematic review. Caries Res.

[ref16] Jin J (2023). Dental caries in adults, adolescents, and children. JAMA.

[ref19] Liu J, Weng Q, Da D, Yao S, Zhang Y, Wu Y (2024). Enhancement of fluoride’s antibacterial and antibiofilm effects against oral Staphylococcus aureus by the urea derivative BPU. Antibiotics (Basel).

[ref20] Manchanda S, Sardana D, Liu P, Lee GH, Li KY, Lo EC (2022). Topical fluoride to prevent early childhood caries: systematic review with network meta-analysis. J Dent.

[ref22] Miller FY, Campus G, Giuliana G, Piscopo MR, Pizzo G (2012). Topical fluoride for preventing dental caries in children and adolescents. Curr Pharm Des.

[ref23] Mohammadi TM, Hajizamani A, Hajizamani HR, Abolghasemi B (2015). Fluoride varnish effect on preventing dental caries in a sample of 3–6 years old children. J Int Oral Health.

[ref24] Munteanu A, Holban AM, Păuna MR, Imre M, Farcașiu AT, Farcașiu C (2022). Review of professionally applied fluorides for preventing dental caries in children and adolescents. Appl Sci.

[ref26] Pieper D, Rombey T (2022). Where to prospectively register a systematic review. Syst Rev.

[ref27] Pontigo-Loyola AP, Mendoza-Rodriguez M, de la Rosa-Santillana R, Rivera-Pacheco MG, Islas-Granillo H, Casanova-Rosado JF (2024). Control of dental caries in children and adolescents using fluoride: an overview of community-level fluoridation methods. Pediatr Rep.

[ref28] Pørksen CJ, Ekstrand KR, Markvart M, Larsen T, Garrido LE, Bakhshandeh A (2023). The efficacy of combined arginine and probiotics as an add-on to 1450 ppm fluoride toothpaste to prevent and control dental caries in children – a randomized controlled trial. J Dent.

[ref30] Shahid M (2017). Regular supervised fluoride mouthrinse use by children and adolescents associated with caries reduction. Evid Based Dent.

[ref31] Sköld UM, Birkhed D, Xu JZ, Lien KH, Stensson M, Liu JF (2022). Risk factors for and prevention of caries and dental erosion in children and adolescents with asthma. J Dent Sci.

[ref32] Twetman S, Petersson L, Axelsson S, Dahlgren H, Holm AK, Källestål C (2004). Caries-preventive effect of sodium fluoride mouthrinses: a systematic review of controlled clinical trials. Acta Odontol Scand.

[ref33] Walsh T, Worthington HV, Glenny AM, Marinho VC, Jeroncic A (2019). Fluoride toothpastes of different concentrations for preventing dental caries. Cochrane Database Syst Rev.

[ref34] Wang Z, Rong W, Xu T (2022). Effect of fluoride varnish in preventing dental caries of first permanent molars: a 24-month cluster randomized controlled trial. Int J Environ Res Public Health.

[ref35] Wasiluk A (2017). Fluoride compounds in dental caries prophylaxis in children and adolescents – review of Polish literature. Przegl Epidemiol.

[ref36] Yang Q, Li F, Ye Y, Zhang X (2025). Antimicrobial, remineralization, and infiltration: advanced strategies for interrupting dental caries. Med Rev.

[ref37] Zhou N, Wong HM, Wen YF, McGrath C (2019). Efficacy of caries and gingivitis prevention strategies among children and adolescents with intellectual disabilities: a systematic review and meta-analysis. J Intellect Disabil Res.

